# How Twitter Can Support the HIV/AIDS Response to Achieve the 2030 Eradication Goal: In-Depth Thematic Analysis of World AIDS Day Tweets

**DOI:** 10.2196/10262

**Published:** 2018-11-22

**Authors:** Michelle Odlum, Sunmoo Yoon, Peter Broadwell, Russell Brewer, Da Kuang

**Affiliations:** 1 Columbia University Medical Center New York, NY United States; 2 Charles E Young Research Library University of California Los Angeles Los Angeles, CA United States; 3 University of Chicago Medicine Chicago, IL United States; 4 Amazon Seattle, WA United States

**Keywords:** community, human rights, social network, infodemiology, infoveillence, Twitter

## Abstract

**Background:**

HIV/AIDS is a tremendous public health crisis, with a call for its eradication by 2030. A human rights response through civil society engagement is critical to support and sustain HIV eradication efforts. However, ongoing civil engagement is a challenge.

**Objective:**

This study aimed to demonstrate the use of Twitter data to assess public sentiment in support of civil society engagement.

**Methods:**

Tweets were collected during World AIDS Days 2014 and 2015. A total of 39,940 unique tweets (>10 billion users) in 2014 and 78,215 unique tweets (>33 billion users) in 2015 were analyzed. Response frequencies were aggregated using natural language processing. Hierarchical rank-2 nonnegative matrix factorization algorithm generated a hierarchy of tweets into binary trees. Tweet hierarchy clusters were thematically organized by the Joint United Nations Programme on HIV/AIDS core action principles and categorized under HIV/AIDS Prevention, Treatment or Care, or Support.

**Results:**

Topics tweeted 35 times or more were visualized. Results show a decrease in 2015 in the frequency of tweets associated with the fight to end HIV/AIDS, the recognition of women, and to achieve an AIDS-free generation. Moreover, an increase in tweets was associated with an integrative approach to the HIV/AIDS response. Hierarchical thematic differences in 2015 included no prevention discussion and the recognition of the pandemic’s impact and discrimination. In addition, a decrease was observed in motivation to fast track the pandemic’s end and combat HIV/AIDS.

**Conclusions:**

The human rights–based response to HIV/AIDS eradication is critical. Findings demonstrate the usefulness of Twitter as a low-cost method to assess public sentiment for enhanced knowledge, increased hope, and revitalized expectations for HIV/AIDS eradication.

## Introduction

### Background

The United Nations’ sustainable development goals (SDGs) seek a holistic and balanced approach to social, economic, and environmental aspects of development [1,2]. The SDGs emphasize the need to advance and complete the objectives set forth by the millennium development goals (MDGs), including a call to end the HIV/AIDS pandemic by 2030 [1,3,4]. An effective response to the pandemic’s end demands critical, dedicated, and sustained action. However, the SDGs [2] have a broad health goal that does not recognize HIV/AIDS as a distinct focus area [1-3]. This is a result of the vertical prioritization of HIV/AIDS during the 2000 to 2015 MDG period [4] that reduced the allocation of resources from other important health issues [1,2,5]. With limited visibility in the post-2015 agenda and lack of additional resources to scale up efforts [1,6], achieving the 2030 eradication goal is of great concern.

The MDGs resulted in the expansion of 15 million people living with HIV/AIDS (PLWH) on life-saving antiretroviral (ARV) drugs [1,3,4,7]. Although the need to scale up and sustain these biomedical solutions is recognized, human rights issues (eg, stigma, marginalization, and discrimination) still serve as pervasive barriers to successful adoption of the goal to end new HIV infections by 2030 [3,8]. However, if HIV/AIDS is not perceived as an ongoing global health emergency, the necessary services and resources to sustain and expand eradication efforts will rapidly diminish. Grassroots activism [8-10] and civil society mobilization [10,11] are critical driving forces for the advancement of human rights and play a major role in the global response to HIV/AIDS [12]. Early in the pandemic, civil society organizations understood the limitations of a solely biomedical focus and need to advance a human rights approach, which resulted in the global scale-up of access to ARVs [3,11]. Further strengthening of the human rights response can support and sustain eradication efforts. According to the Joint United Nations Programme on HIV/AIDS (UNAIDS), strong civil society engagement is critical to the eradication of HIV/AIDS [11,12]. Ongoing human rights–related efforts, which include giving voices to PLWH and empowering marginalized populations, are essential for the successful mobilization of treatment and prevention resources [10]. In fact, the SDGs demand grassroots activism [4,8,9,11] and call for a greater investment and support of civil society to achieve the eradication goal [1,2,13]. Effective civil society activism [8,11] must engage advocacy networks, private and public institutions, and global policy makers to advance the 2030 campaign [14]. However, the life cycle of activism [8,9,12] and its ability to sustain influence and engage citizens is one of the greatest challenges and opportunities to the eradication of HIV/AIDS. Furthermore, the success of civil society activism [8,9] lies in exploration of public sentiment to guide the effective exchange of information for improved knowledge, increased hope, and revitalized expectations [11,12].

### Objective

The utilization of social networking sites (SNSs) for civil society activism [8,9] is a promising approach to help sustain and drive HIV/AIDS-related social movements. SNSs, such as Twitter, have played a vital role in the organization of global movements [15]. Twitter is a very powerful and popular microblogging communication tool [16]. Users share information through 280-character messages called tweets. Information is disseminated through direct messages or the forwarding (retweeting) of messages for broad propagation [15,17]. In the field of infodemiology and digital surveillance (infoveillance), Twitter is effectively used to predict disease outbreaks, including the flu and HIV, and has informed a variety of public health efforts [18-20]. Public sentiment, expressed in tweets, provides a wealth of information to be used by public health professionals, politicians, governmental entities, activists, and computer scientists; to engage in purposeful discussions; and to play active roles on a variety of topics [11,16,17]. Moreover, Twitter has the capability to identify health beliefs and to support interventions and health campaigns for improved motivation and behavior. Twitter is also used effectively to assess and address health information needs during disease outbreaks, such as Ebola [15,17,21,22]. With more than 645 million registered users and the distribution of more than 58 million tweets daily, Twitter is a reliable source to track public sentiment and guide discussions for effective health awareness campaigns [14,17,21]. Public motivation is essential to sustain the global HIV/AIDS response and to achieve our global eradication goals. This study aimed to demonstrate the use of Twitter to explore HIV/AIDS public sentiment at 2 separate time points [15] to help guide social movements in support of HIV/AIDS eradication efforts, which are guided by UNAIDS core action principles of a comprehensive HIV/AIDS response [23] and commitments necessary to reach the 2030 goal of HIV prevention, treatment, care, and support. World AIDS Day, held on first of December every year, provides an ideal opportunity to assess public sentiment as people unite worldwide to support PLWH, to honor those lost, and to combat HIV/AIDS [24]. Our study provides a unique and in-depth analysis of World AIDS Day tweets in 2014 and 2015.

## Methods

### Tweet Corpus

During the World AIDS Days of December 1, 2014, and December 1, 2015, tweets about HIV/AIDS were collected from Twitter via a Google Chrome–based version of NCapture, a Web crawler that captures internet-based text [25]. The streaming application programming interface allowed for the capture of a limited sample of all tweets (eg, 18,000 tweets per 15 min) [25]. To overcome the challenges of time and amount limits, tweets were collected in 15-min intervals for a representative sample [25]. Keywords were used for searching tweets that mentioned HIV/AIDS (eg, #HIVtreatment, #HIVservices, #HIVprogramming, and #HIVprevention). Additional data elements collected were time stamps, content, and geographical locations from IP addresses, usernames, message type (unique or retweet), and followers (number of disseminated) [25]. English language tweets were included in the analysis, with 39,940 unique tweets (10,027,038,772 users) in 2014 and 78,215 unique tweets (33,370,938,359 users) in 2015.

### Natural Language Processing

Natural language processing was conducted to identify and depict topics of collected tweets about HIV/AIDS. Tweets were cleaned and transformed to a vector form and N-gram [25]. An N-gram is a subsequence of N items in a given sequence from characters to words. The N-gram method was used to compute a tweet term–frequency dictionary [25]. Notepad++v7.2.2 developed by Don Ho and Weka 3.7 developed by the University of Waikato in New Zealand both are open source software reduced the dimensionality for the algorithmic processing of the data. Snowball stemmers were used to apply the stemming algorithm, Porter algorithm, an affix-removal approach, which was applied through Weka [25]. To further remove dimensionality, stemming was conducted to identify a word root and to remove suffixes and prefixes. Tweet topics were detected and summarized through descriptive statistics (eg, frequency, defined as the aggregate number of times a topic is tweeted), classification, visualization, and clustering.

### Hierarchical Rank-2 Nonnegative Matrix Factorization Algorithm and Rank-2 Nonnegative Matrix Factorization

**Figure 1:**
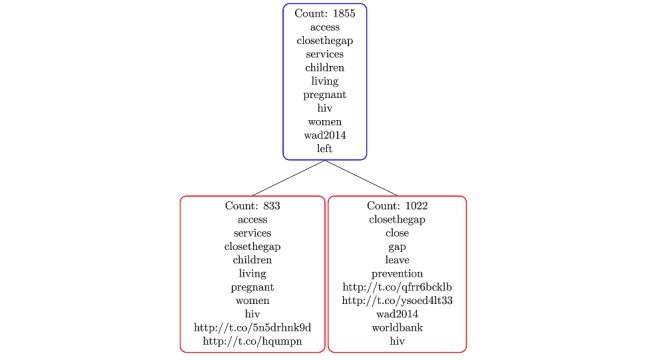
An illustration of a nonleaf node N (Blue) and its 2 potential children split L (left) and R (right) in red into leaf nodes (primary analysis phase).

Clustering, the process of grouping a set of words into classes of similar topics, was conducted [26]. Hierarchical rank-2 nonnegative matrix factorization (HierNMF2) was used to determine the semantic organization of tweeted words [26,27]. Data were analyzed with the Python 2.7 software by the Python Software Foundation and were treated independently and analyzed separately by years. Tweet topic clusters were generated by HierNMF2 and visualized into tree nodes for 2014 and 2015 data to illustrate the topic structure. The HierNMF2 used for clustering generated a hierarchy of tweet themes into binary trees [26]. The hierarchy is automatically detected but does not always result in a balanced tree (Figure 1). A node-splitting rule was also employed to determine tree nodes to split from the original binary nodes. This methodology allows for the determination of tree structures. Data for each time point (2014 and 2015) were split into 2 clusters, creating a binary tree [26]. Rank-2 nonnegative matrix factorization (NMF) was applied to generate the hierarchical tree structure. Each cluster created nonleaf nodes (nln). A score was computed for each nln using rank-2 NMF [26,27]. The nln with the highest scores were then split into leaf nodes (ln) of 2 or more well-separated topics. Specific details of algorithm development have been previously published by a coauthor (DK) [26,27].

### Thematic Analysis

A coding framework was used to interpret and explore the data and to identify perceptions of the HIV/AIDS pandemic. Tweet hierarchy raw data clusters were characterized based on words in each cluster of nln and ln by subject matter experts (MO and RB; Figure 1). Hierarchical cluster analysis occurred iteratively, based on cluster content (eg, *cluster words*: “fight, today, support, cure, love”; *associated theme*: Combat HIV/AIDS). Major themes were refined through review and discussion, with our experts (MO and RB), to shape the final coding structure. Themes were organized based on UNAIDS core action principles (eg, *theme*: Combat HIV/AIDS; *principle*: Safeguard Human Rights) for guiding a comprehensive response to HIV/AIDS (Table 1). Themes were then categorized according to the commitments necessary to reach the SDG’s 2030 goal [4]: Prevention, Treatment or Care, and Support (eg, *theme*: Combat HIV/AIDS; *principle:* Safeguard Human Rights; *commitment area*: Support).

Ethics committee approval was not required for Twitter analysis because tweets are deidentified with no identifiable information obtained.

**Table 1:** Thematic analysis of 2014 and 2015 World AIDS Day tweet hierarchy.

Hierarchy—degree levels in hierarchy		Themes			World AIDS Day 2014	World AIDS Day 2015
2014	2015	Comprehensive Global Response to HIV/AIDS			Representative example tweets	
		UNAIDS^a^ Core Actions		Prevention, Treatment or Care, and Support		
1⁰	2⁰	Prevention	Decrease Vulnerability of Acquiring HIV/AIDS	Informational Resources from Governmental Organizations	infographics, AIDS.gov	white house, AIDS.gov
4⁰	5⁰	Prevention	Decrease Vulnerability of Acquiring HIV/AIDS	Halt Infections	accessible, affordable, vaccine partnerships	epidemic, averted
5⁰	4⁰	Prevention	Decrease Vulnerability of Acquiring HIV/AIDS	AIDS-Free Generation	Close the gap	aids free gen, children
4⁰	—	Prevention	Decrease Vulnerability of Acquiring HIV/AIDS	Prevent HIV/AIDS	aids prevention, HIV facts	—
5⁰	3⁰	Treatment or Care	Access to Services	Treatment for HIV/AIDS	Access, children, pregnant women services, closing the gap	treat, people
4⁰	1⁰	Treatment or Care	Access to Services	Save Lives: Infected and At Risk	cure	treatment for all
—	3⁰	Treatment or Care	Access to Services	Impact of the HIV/AIDS Pandemic	—	millions, lives-end, children, can avert, infections-save, world
1⁰	1⁰	Treatment or Care	Expand Programs	Efforts for Targeted HIV/AIDS Eradication	facts, wipe homophobia, today	save, join, treatment for all
3⁰	6⁰	Treatment or Care	Expand Programs	Fast Track the End of the Pandemic	today, end, epidemic	unaids, fast track, treatment, response, assessing
4⁰	2⁰	Support	Safeguard human rights	Spread HIV/AIDS awareness	cure, love, sending, support, fight, amfar^b^	awareness, spread, end aids
5⁰	3⁰	Support	Safeguard human rights	Recognition of the Pandemic	unaware, United States, hiv	Aware, disease
4⁰	1⁰	Support	Safeguard human rights	Honor People Living with HIV/AIDS (PLWH)	honor, memory, continuing, lost, affected	living, people, positive, statement
4⁰	2⁰	Support	Safeguard human rights	Expression of HIV/AIDS Solidarity and Consciousness	wear, ribbon, close the gap	tee shirt ribbon
2⁰	5⁰	Support	Safeguard human rights	Combat HIV/AIDS	fight, today, support, cure, love	fight, helping
3⁰	2⁰	Support	Safeguard human rights	Support PLWH	sending, support, fight, cure	support, living, and people
4⁰	2⁰	Support	Partnerships and Alliances	Commitment to End the Pandemic	renew, vow, longer	statements, discrimination, make
2⁰	5⁰	Support	Partnerships and Alliances	Celebrities or Industries	mac^c^ cosmetics viva glam, mac aids Fund	Kasper, rappers, Victoria Beckham
2⁰	2⁰	Support	Eliminate Stigma and Discrimination	Reduce Stigma	Lgbt^d^, facts	stigma, end
—	3⁰	Support	Eliminate Stigma and Discrimination	Discrimination of PLWH	—	discrimination, living, people

^a^UNAIDS: Joint United Nations Programme on HIV/AIDS.

^b^amfAR: American Foundation of AIDS Research.

^C^MAC: Make-up Art Cosmetics.

^d^LGBT: lesbian, gay, bisexual, and transgender.

## Results

### Response Frequencies

**Figure 2:**
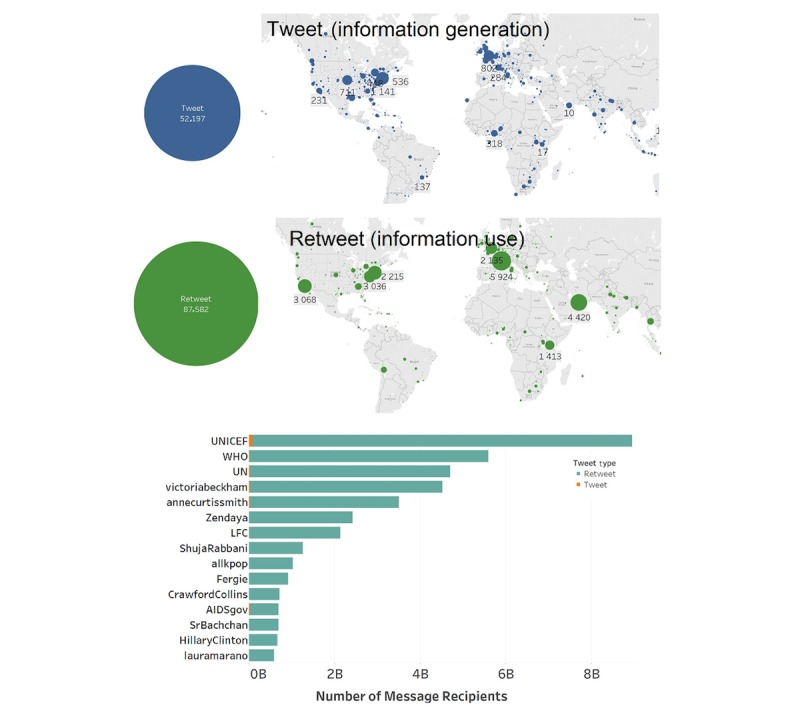
World AIDS Days 2014 and 2015: geographic location and top disseminators.

The geographic spread of HIV/AIDS-related tweets on World AIDS Days 2014 and 2015 spanned the globe (Figure 2). Top disseminators were United Nations agencies followed by celebrities, including singers, models, actors, and US governmental organizations and political figures.

**Figure 3:**
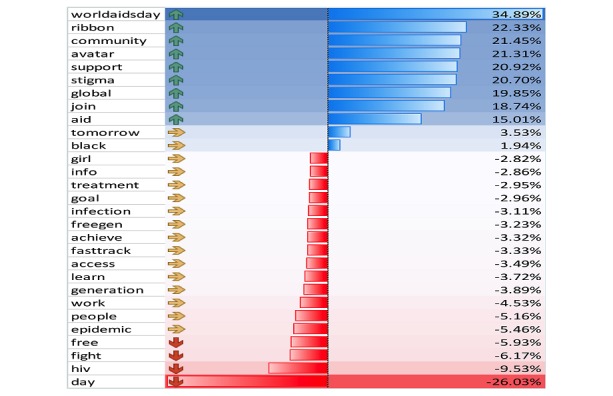
Shift in HIV/AIDS public sentiment: World AIDS Day tweets from 2014 to 2015.

Topics tweeted 35 times or more were aggregated. Response frequencies were generated and compared for increased and decreased frequency between 2014 and 2015. Results show increased frequency in tweets associated with an integrative approach to HIV prevention, treatment, and care (eg, community). An increase was also observed in the frequency of tweets associated with the recognition of barriers to HIV/AIDS eradication (eg, stigma). A moderate decline was observed in tweets associated with ending the epidemic (eg, fast track) and the provision or utilization of services (eg, access). A significant decrease in tweet frequency associated with combating the epidemic (eg, fight) was also observed (Figure 3).

### Hierarchical Clusters of Tweets

**Figure 4:**
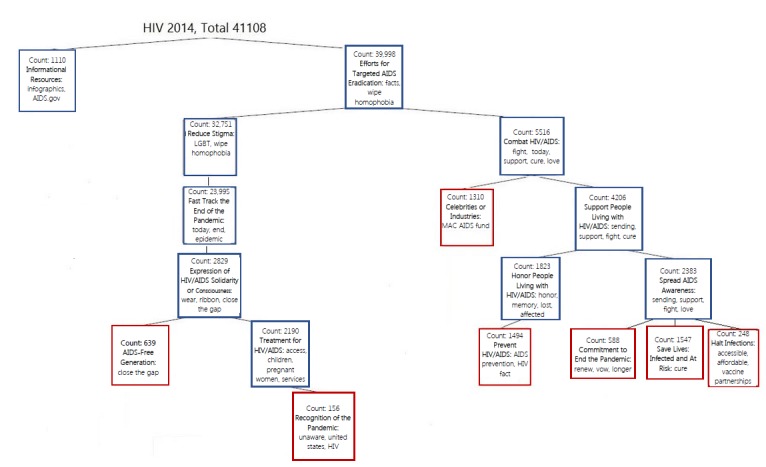
World AIDS Day 2014 tweet hierarchy.

World AIDS Day 2014 tweets were clustered into a binary tree: Informational Resources from Governmental Organizations versus Efforts for Targeted HIV/AIDS Eradication. The tweet cluster of *Efforts for Targeted HIV/AIDS Eradication* led to 2 nln. First, Reduce Stigma (nln) followed in order of descent by (1) Fast Track the End of the Pandemic (nln), (2) Expression of HIV/AIDS Solidarity and Consciousness (nln), (3) AIDS-Free Generation (ln), (4) Treatment for HIV/AIDS (nln), and (5) Recognition of the Pandemic (ln). Second, Combat HIV/AIDS (nln) followed in order of descent by (1) Celebrities or Industries (ln), (2) Support PLWH (nln), (3) Honor PLWH (nln), (4) Prevent HIV/AIDS (nln), (5) Spread HIV/AIDS Awareness (nln), (6) Commitment to End the Pandemic (ln), and (7) Save Lives: Infected and At Risk and Halt Infections (ln; Figure 4).

**Figure 5:**
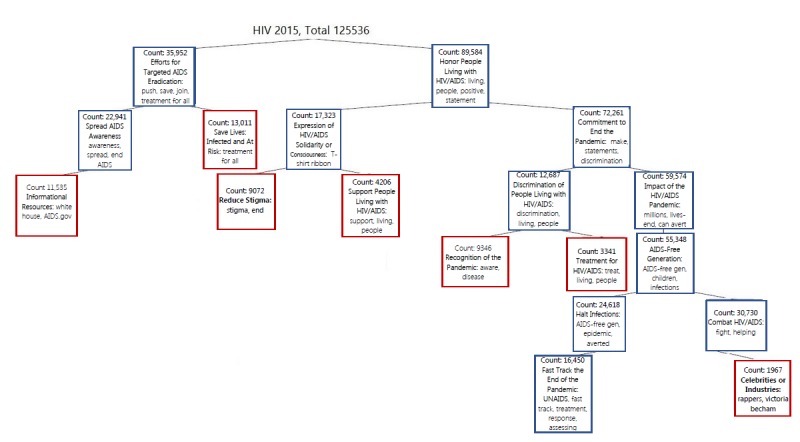
World AIDS Day 2015 tweet hierarchy.

World AIDS Day 2015 tweets were clustered into a binary tree: Efforts for Targeted AIDS Eradication versus Honor PLWH. The tweet cluster of *Efforts for Targeted HIV/AIDS Eradication* followed in order of descent by (1) Save Lives: Infected and At Risk (ln), (2) Spread HIV/AIDS Awareness (nln), and (3) Informational Resources from Governmental Organizations (ln). The tweet cluster of *Honor PLWH* followed in order of descent by (1) Expression of HIV/AIDS Solidarity (nln), (2) Reduce Stigma (ln), and (3) Support PLWH (ln) versus Commitment to End the Pandemic (ln) followed in order of descent by (4) Discrimination of PLWH (nln), (5) Recognition of the Pandemic (ln), and (6) Treatment for HIV/AIDS (ln) versus Impact of HIV/AIDS Pandemic (ln) followed in order of descent by (7) AIDS-Free Generation (nln), (8) Halt Infections (nln), and (9) Fast Track the End of the Pandemic (nln) versus Combat HIV/AIDS (nln) followed in order of descent by (10) Celebrities or Industries (ln; Figure 5).

### Thematic Analysis

#### Prevention

In Prevention, 1 overarching theme emerged. The theme was *Decrease Vulnerability of Acquiring HIV/AIDS* and included the individual themes of (1) Informational Resources from Governmental Organizations, (2) Halt Infections, (3) AIDS-Free Generation, and (4) Prevent HIV/AIDS (2014 only).

#### Treatment or Care

In Treatment or Care, 2 overarching themes emerged: *Access to Services*, including the individual themes of (1) Treatment for HIV/AIDS (2015 increase), (2) Save Lives: Infected and At Risk (2015 increase), and (3) Impact of the HIV/AIDS Pandemic (2015 only), and *Expand Programs*, including the individual themes of (1) Efforts for Targeted HIV/AIDS Eradication and (2) Fast Track the End of the Pandemic (2015 decrease).

#### Support

In Support, 3 overarching themes emerged: *Safeguard Human Rights*, including the individual themes of (1) Spread HIV/AIDS Awareness (2015 increase), (2) Recognition of the Pandemic (2015 increase), (3) Honor PLWH (2015 increase), (4) Expression of HIV/AIDS Solidarity and Consciousness (2015 increase), (5) Combat HIV/AIDS (2015 decrease), and (6) Support PLWH; *Partnerships and Alliances*, including the individual themes of: (1) Commitment to End the Pandemic (2015 increase) and (2) Celebrities or Industries (2015 decrease); and *Eliminate Discrimination and Stigma*, including the individual theses of (1) Reduce Stigma and (2) Discrimination of PLWH (2015 only; Table 1).

## Discussion

### Principal Findings

HIV/AIDS is one of the greatest challenges to sustainable social, economic, and civil society development and affects all sectors of our society [1,6,11,28]. Strong civil engagement to drive social change remains critical to HIV/AIDS eradication [3,11]. This study demonstrated how the analysis of social media data, specifically Twitter, could inform purposeful discussions for effective civil society engagement. Our thematic analysis of the World AIDS Days 2014 and 2015 Twitter hierarchies identified public sentiment on a variety of human rights– and biomedical-related topics. The majority of themes fell primarily under Support, followed by Treatment or Care, and then Prevention. In fact, the theme *Prevent HIV/AIDS* was present only in 2014. Prevention words were not present in the 2015 hierarchy. An absence of this theme may reflect treatment as prevention because of the uptake of pre-exposure prophylaxis. This may indicate the need for purposeful discussion, as prevention needs are not only biomedical. Human rights–based prevention approaches will ensure a more permanent and sustainable solution to achieve HIV eradication. The future cost of daily ARVs in developing countries is not sustainable [7]. Therefore, the revitalization of human rights–based initiatives is a priority. Early in the pandemic, human rights–based approaches focused on health equity and worked with populations living with and affected by HIV to expand ARVs in the pipeline to halt the spread of HIV [7,29]. The need exists for the constant engagement of civil society [7,11,12] in purposeful discussions to revitalize prevention campaigns [14].

Under Treatment or Care, the theme of *Impact of the Pandemic* was seen only in 2015. The emergence of this theme may reflect a heightened awareness or the recognition of the pandemic’s effects on those infected and affected. The theme of *Efforts for Targeted HIV/AIDS Eradication* remained as a primary level theme in both years. In 2014, tweets discussed the elimination of homophobia and knowledge about HIV/AIDS. Tweets from 2015 discussed treatment, saving lives, and joining efforts. Although discrimination was present in 2015, the recognition of homophobia was seen only in 2014. This may indicate a need for purposeful discussion, particularly on the importance of supporting key populations [30]. The theme of *Fast Track End of the Pandemic* decreased 3 levels on the hierarchy in 2015. In 2014, tweets discussed the end of the pandemic, an indication of action and words of excitement. In 2015, tweets called for an assessment of the HIV/AIDS response. With 2015 marking the end of the MDGs, this may reflect our shortfall of the 2015 goals and the need for improved effort under the SDGs [3].

In 2015, all themes increased on the hierarchy under Support, except *Combat HIV/AIDS*, which decreased 3 levels. This theme comprises action words necessary to end the pandemic, including fight and support. The shift may be an indicator of lost enthusiasm, similarly discussed under Treatment or Care. It is also important to note the absence of such words based on our response frequency analysis. Decreases were observed in tweets associated with efforts to eradication (eg, AIDS-free generation). This may further reflect the need to re-engage the public in purposeful discussions and to reinvigorate grassroots efforts [10]. The theme *Reduced Stigma* remained a second-level tweet in 2015, with 2014 data acknowledging the lesbian, gay, bisexual, transgender, and queer community and 2015 data discussing the need to end stigma [8,11,30,31]. Maintaining this discussion is critical, as key populations are disproportionately affected by HIV/AIDS. Key populations are criminalized, marginalized, and plagued with stigma [32] and contribute to HIV risk [8,31]. Essential services for prevention are often unavailable to these groups [30]. Furthermore, HIV-associated stigma also contributes to poor HIV treatment and care access [30]. The theme of *Discrimination of PLWH* emerged in 2015. This may potentially reflect the November 2014 UNAIDS Fast-Track Strategy report, with zero discrimination recognized as the main target to end HIV/AIDS [3]. However, in the 2015 data on recognition of stigma and discrimination, tweets were not focused on key population or high-risk groups [3,8,30]. Results indicate the need for ongoing discussion on such barriers, as civil society plays a major role in the support of marginalized populations [11,12,31].

Hierarchical differences also revealed absence of tweets in 2015 mentioning women. This is of great concern; issues of gender minorities must be addressed or eradication efforts may be thwarted. The focus on key populations does very little to change gender inequality [33]. Women are only considered key when they are pregnant, nursing, or members of other high-risk groups (eg, sex workers) [3]. This approach, which neglects women living with HIV and other vulnerable women, fails to transform norms, beliefs, and perceptions about women’s rights to health and well-being [33]. In fact, 2014 tweets mentioned women only in the context of motherhood. Women comprise over 60% of PLWH globally [33]. The biological and social factors that make them most vulnerable to HIV infection must be addressed. Persistent discussions about women’s rights remain critical for eradication.

### Strengths and Limitations

The generalizability of this study is limited because of the English-only analysis and the use of 1 SNS (ie, Twitter) [15]. We did not include composed tweets, only those disseminated through tweeting or retweeting. For a more in-depth analysis of social media data, future studies should explore other SNSs and analyses in other languages. Twitter’s global and pervasive spread of information can support civil society engagement [15,22]. With the limitation of our dataset to data collection on 1 day out of each year and data analysis of only 2 consecutive years, our study lacked the power to assess trends. Future studies should include data collection days before and after World AIDS Day for multiple years to apply dynamic topic modeling to the data to effectively monitor change over time. Due to the randomness of tweets and potential of specific contents to go viral, generalizations of missing themes over time may be a consequence of the nature of Twitter and not actual changes in perceptions. Nevertheless, Twitter data have the potential to supplement traditional survey methods and provide tremendous insight into understanding public beliefs and sentiment.

### Conclusions

Our ambitious targets are critical to the pandemic and are possible with the support of technology and social media outlets such as Twitter [15,16]. Civil society’s human rights–based approaches and responses can be limited by material resources [11,12]. The low cost and ubiquitous spread of information through SNSs can diminish such barriers. The vision of zero new HIV infections, zero discrimination, and zero AIDS-related deaths must be transformed into tangible milestones and end points [1,6], and social media can help support these efforts [3,16,21,22].

Our study’s demonstration of Twitter utilization to explore HIV/AIDS public sentiment can guide targeted social movement campaigns aimed to address grassroots level barriers and heighten public motivation necessary to drive eradication. We also demonstrated the feasibility of the use of cost-effective social networking technologies to identify health-related communication and the utilization of such platforms to support improved outcomes. In fact, with the ever-increasing amount of social media data and the unique and refined analytic approaches such as ours, HIV/AIDS researchers and global health professionals will soon be able to build upon and enhance their methods, to accurately monitor and support a variety of HIV-related issues, and outcomes.

## Abbreviations

ARV: antiretroviral
HierNMF2: hierarchical rank-2 nonnegative matrix factorization
ln: leaf nodes
MDG: millennium development goal
nln: nonleaf nodes
NMF: nonnegative matrix factorization
PLWH: people living with HIV/AIDS
SDG: sustainable development goals
SNS: social networking site
UNAIDS: Joint United Nations Programme on HIV/AIDS
